# Comment on: “Effects of Exercise Training Interventions on Executive Function in Older Adults: A Systematic Review and Meta‑analysis”

**DOI:** 10.1007/s40279-020-01369-7

**Published:** 2020-10-31

**Authors:** Philipp Zimmer, Florian Javelle, Amit Lampit

**Affiliations:** 1 Institute for Sport and Sport Science, Division of Performance and Health (Sports Medicine), TU Dortmund University, Otto-Hahn-Straße 3, 44227 Dortmund, Germany; 2 Institute for Molecular and Cellular Sports Medicine, German Sport University Cologne, Cologne, Germany; 3 Academic Unit for Psychiatry of Old Age, Department of Psychiatry, The University of Melbourne, Melbourne, Australia; 4 Department of Neurology, CHARITÉ-Universitätsmedizin Berlin, Berlin, Germany

Dear Editor,

As the evidence base for the efficacy of physical exercise for cognition in older adults is mounting, efforts to detect the most efficacious exercise settings are critical for clinical translation and guidelines development. Chen et al. [1] conducted a systematic review and a series of subgroup meta-analyses aiming to investigate possible moderators of exercise effects on executive functions in cognitively healthy people and those with mild cognitive impairment. This is undoubtedly a critical question to move the field forward, not only for clinical translation but also for rigorous trial design, and particularly suitable for meta-analysis. However, we believe that a number of critical methodological flaws raise serious concerns regarding the reliability of the findings reported by Chen et al. [1].

First and perhaps most fundamentally, Chen et al. [1] have substantially overestimated the precision of their findings due to counting multiple (and thus non-independent) effect sizes as independent units of analysis. As Table 3 in Chen et al. [1] indicates, the 33 articles included in the analysis reported 107 effect sizes. When multiple outcomes (in this case, cognitive tests) or subgroups are nested within a single study, or reported across several manuscripts (e.g., three included articles by Gothe et al. [2–4]), the included effect sizes are not independent since they provide information on the same participants. This is a common problem in meta-analysis but various solutions have been proposed to handle dependency [5] and have been applied in meta-analyses of exercise effects on cognition [6, 7]. Yet Chen et al. [1] treated each effect size independently, leading to (a) overestimation of sample size from a total of *n* = 3008 (Table 2) to 7023 in the overall model, (b) underestimation of standard error and thus overestimation of precision, and (c) assigning excessive weight to studies that reported multiple outcomes regardless of their actual sample size, which bias not only the pooled effect estimates but also investigations of small-study effect (‘publication bias’). Although Chen et al. [1] did not provide forest plots, it is safe to assume, for example, that the sample in Gothe et al. [2–4] was counted as seven separate samples, while Albinet et al. [8] was counted eight times. While such cases of ‘double-counting’ may occur sporadically, perhaps due to oversight [9], these are so pervasive across Chen et al.’s analyses [1] that the results cannot be even remotely reliable.

Second, all the analyses reported by Chen et al. [1] are based on a fixed effect model, which is generally inappropriate for pooling such a heterogeneous set of studies, cannot be generalized beyond the scope of the included studies and typically overestimates the precision of the mean effect [10, 11]. Conceptually, a fixed effect meta-analysis assumes that all studies are assessing the same underlying effect, and thus any variation between studies is attributed to chance rather than important differences between studies [10, 11]. Computationally, this model weights studies solely on the basis of their variance (inverse-variance weighting) and ignores between-study heterogeneity. The studies included in Chen et al. [1] are drawn from the literature and differ in ways that can affect the results, such as type of population, intervention, outcomes, controls, and risk of bias. Investigating how these differences related to the efficacy of exercise was cited as one of the two aims of Chen et al.’s work [1]. Unsurprisingly, the overall analysis (Table 3) revealed substantial heterogeneity in true effects between studies, which was unlikely to be due to chance given the results of the Cochran test [*Q*(106) = 260.09, *p* < 0.01] [1]. While meta-analysis based on inverse-variance weighting may often perform well even in the presence of heterogeneity, the conditions under which such arguments are made [12] are unlikely to apply for the diverse set of studies in Chen et al. [1].

Third, it is not clear to us why the authors chose to include only studies published since 2003, i.e., after the publication of Colcombe and Kramer’s seminal meta-analysis in this area [13]. In their discussion, Chen et al. [1] state that their goal was to update the findings since Colcombe and Kramer’s work [13]. However, excluding studies that were included in previous reviews defies not only the logic of an update but also basic systematic review practices, mainly those recommended by PRISMA [14] and the Cochrane Handbook [15]. To that end, we find it surprising that although Chen et al. [1] claim to follow these widely accepted guidelines, their methods and reporting standards (e.g., in terms of reproducibility) are closer to those of Colcombe and Kramer [13] than to those recommended by PRISMA. Notwithstanding the importance of Colcombe and Kramer’s original meta-analysis [13], methods and standards in the field have developed considerably over the past 17 years, and basic flaws such as those described here are no longer tenable.

Like many in the exercise science and sports medicine community, we were encouraged by Chen et al.’s [1] positive and interesting results. We do, however, note that the major flaws in this article are at the level of basic meta-analysis principles rather than opinions on statistical modelling. In light of relentless efforts to improve the quality of studies and optimize the effects of exercise on body and mind across the lifespan, we advise against the use of this meta-analysis to inform study design or clinical practice, and hope to see much more robust trials and systematic reviews in the future.
